# Muscle proportionality: The proportionality of skeletal muscle before and after intervention

**DOI:** 10.1186/1550-2783-12-S1-P51

**Published:** 2015-09-21

**Authors:** Brian A Jones, Robert T Davidson

**Affiliations:** 1 Health and Sport Sciences Division, Missouri Baptist University, St. Louis, MO, 63141, USA; 2 Nutrition and Human Performance, Logan University, Chesterfield, MO, 63017, USA

## Background

There are many types of intervention that can lead to a change in lean body mass including: hormone replacement therapy, aging, menopause, strength training, aerobic exercise, extreme weight loss interventions (gastric bypass surgery), and starvation. Lean body mass is a metabolically active tissue which is involved in several key functions in the body, including: locomotion, basal metabolic rate, and strength. The purpose of this study was to assess the proportionality of changes of regional lean body mass (arms, trunk and legs) before and after intervention.

## Methods

A systematic search was conducted utilizing these key phrases, "muscle proportionality", "regional muscle proportionality", and "DXA regional muscle studies". Also, each study had to fit within the search criteria which included; DXA regional lean body mass before and after intervention randomized controlled studies. After the preliminary search was conducted, a total of 10 studies fit the search criteria for this study. Initial versus final regional lean body mass was plotted and linear regression R² for total as well as, regional lean body mass (LBM) changes was determined.

## Results

Muscle proportionality was linearly correlated in three specific regions of the body including: arms (r² = 0.94, p < 0.0001), legs (r² = 0.97, p < 0.0001), and trunk (r² = 0.89, p < 0.0001). Total body muscle proportionality was also linearly correlated (r² = 0.99, p < 0.0001).

## Conclusion

In studies utilizing no or whole body weight training, muscle change - gain or loss - appears to occur proportionately to where it was before the intervention. This data could prove beneficial for healthcare professionals when designing nutrition protocols and assessing lean body mass change over time.

